# The Impact of Pre-Operative Aneurysm Diameter on Mortality After Standard and Complex Endovascular Aortic Repair

**DOI:** 10.31083/RCM47199

**Published:** 2026-01-22

**Authors:** Petroula Nana, George Apostolidis, José I. Torrealba, Giuseppe Panuccio, Christian-Alexander Behrendt, Tilo Kölbel

**Affiliations:** ^1^German Aortic Center, Department of Vascular Medicine, University Medical Center Eppendorf, 20251 Hamburg, Germany; ^2^Vascular and Endovascular Surgery, Asklepios Clinic Wandsbek, Asklepios Medical School Hamburg, 22043 Hamburg, Germany

**Keywords:** aorta, aneurysm, diameter, endovascular aortic repair, EVAR, mortality

## Abstract

**Background:**

Various anatomical factors have been related to mortality after endovascular aortic aneurysm repair (EVAR). This systematic review investigated the impact of the pre-operative maximum aortic aneurysm diameter on mortality after standard and complex EVAR.

**Methods:**

The Preferred Reporting Items for Systematic Reviews and Meta-analyses (PRISMA) guidelines were followed to search the MEDLINE, EMBASE, via Ovid and CENTRAL databases, until 31st July 2025. Randomized controlled trials and observational studies were eligible if they were published between 2015 and 2025 and reported on the association of the pre-operative maximum aortic aneurysm diameter with a 30-day and midterm mortality follow-up in standard and complex EVAR patients. The Newcastle-Ottawa Scale assessed the risk of bias. The primary outcome was the impact of the pre-operative maximum aortic aneurysm diameter on 30-day mortality after standard and complex EVAR.

**Results:**

From 1182 studies, 25 were included; 19 reporting on standard (130,476) patients and six on complex EVAR (14,097) patients. A significant heterogeneity in terms of maximum pre-operative aortic aneurysm diameter threshold to identify larger aneurysms was detected. Regarding standard EVAR, eight studies evaluated the impact of the pre-operative maximum abdominal aortic aneurysm (AAA) diameter on 30-day mortality (smaller: 0.3–13.2% vs. larger: 0.7–20.8%) with conflicting outcomes. Four studies (4/8 studies; 50%) concluded that a larger diameter was related to higher 30-day mortality in patients with standard EVAR, while four showed no statistical significance. Two out of five standard EVAR studies that investigated the pre-operative AAA diameter as an independent predictor for 30-day mortality confirmed this finding. During the mid-term follow-up, ten studies showed that the pre-operative maximum AAA diameter was independently related to mortality after standard EVAR. In complex EVAR, four out of six studies showed that the 30-day mortality was higher (smaller: 0.5–7.0% vs. larger: 4.0–15.0%) in larger aortic aneurysms, including juxta-, para-, supra-renal, and thoracoabdominal aortic aneurysms. Four out of five (80.0%) studies showed that a larger diameter was an independent predictor for follow-up mortality after complex EVAR.

**Conclusions:**

The pre-operative aortic aneurysm diameter seems to be related to mortality after standard or complex EVAR. However, the impact of the pre-operative aortic aneurysm diameter on mortality seems to be more prominent in complex EVAR cases, with 80% of studies confirming this finding.

## 1. Introduction

Endovascular aortic aneurysm repair (EVAR) has been established as the preferred treatment option in high-risk patients with infrarenal abdominal aortic aneurysm and suitable anatomy [1, 2, 3]. Similarly, complex EVAR with fenestrated and branched devices is recommended as the first-line treatment in patients with aortic aneurysms affecting the renovisceral aorta and moderate to high risk for peri-operative complications [1]. The main benefit of both standard and complex EVAR is the associated lower short-term mortality and morbidity compared to open surgical repair while conflicting evidence exists regarding EVAR’s long-term survival benefit, which seems to be lost after the mid-term follow-up, despite the low aneurysm-related mortality [2, 3, 4, 5, 6].

Patients’ high-risk profile is the main contributor to higher mortality after standard and complex EVAR; with coronary artery disease, peripheral arterial disease, chronic kidney and chronic obstructive pulmonary disease being related to worse survival [7, 8]. However, a variety of other parameters has been detected to affect EVAR clinical outcomes, including age, sex, proximal and distal sealing zones and extent of the disease [9, 10, 11]. Regarding the role of anatomy on standard EVAR, previous studies showed that hostile proximal neck characteristics and larger abdominal aortic aneurysm (AAA) diameter may be related to worse technical and clinical outcomes during follow-up, especially for the female population [9, 12, 13]. Recent data on complex aortic aneurysms, including juxtarenal, pararenal, and thoracoabdominal aneurysms (TAAA), showed that patients with larger pre-operative aneurysm diameter were at increased risk for mortality at 30-days and mid-term follow-up, arising questions on the current diameter thresholds indicating treatment [14, 15].

Thus, this systematic review aimed to investigate the available literature on the impact of the pre-operative aortic aneurysm diameter on standard and complex EVAR mortality at 30-days and further follow-up (beyond 30 days).

## 2. Methods and Materials 

### 2.1 Eligibility Criteria 

**Fig. 1.**
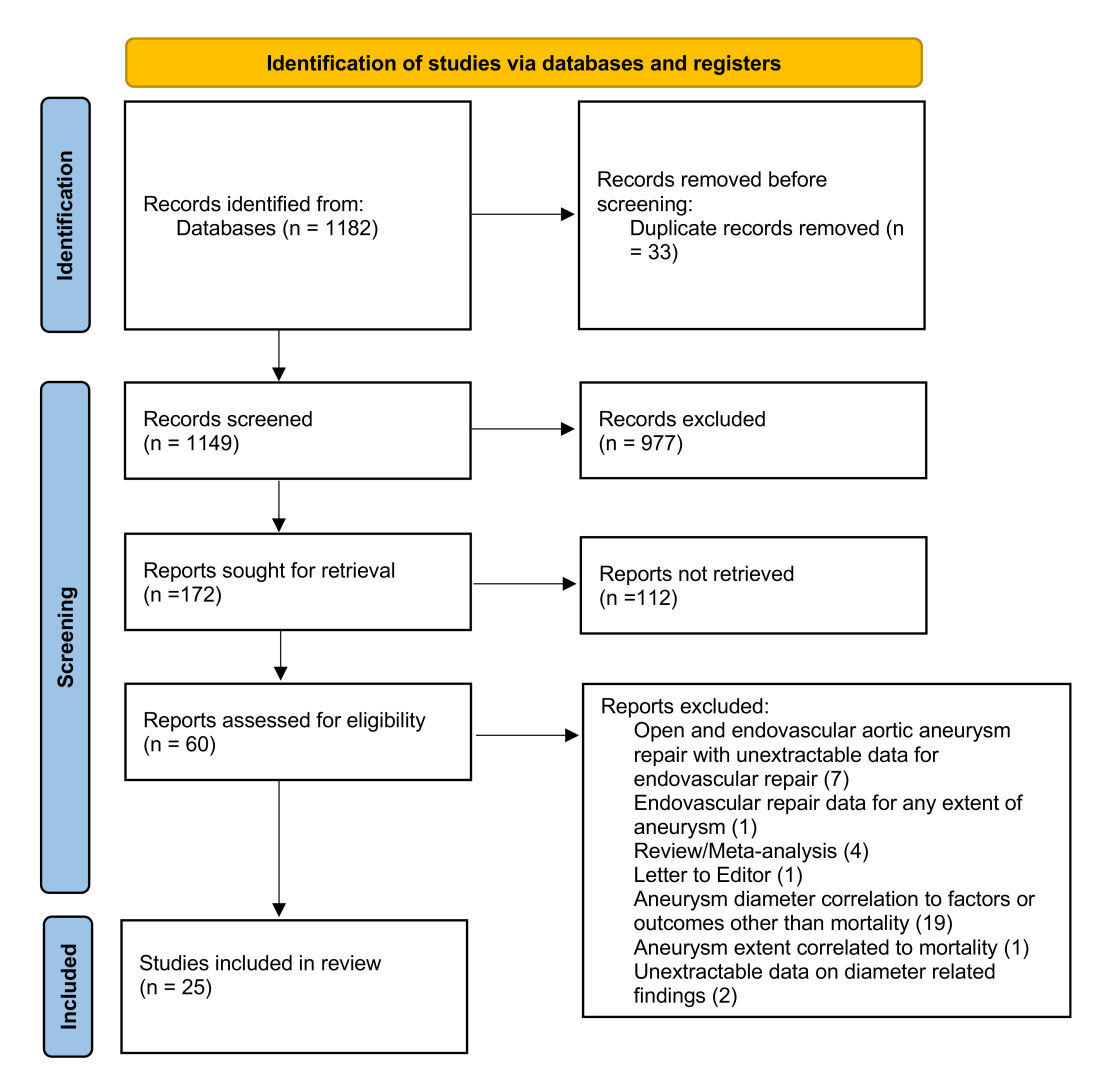
**The PRISMA 2020 flow chart depicting the study selection process**. PRISMA, Preferred Reporting Items for Systematic Reviews and Meta-analyses.

A systematic review of the English medical literature following a pre-defined methodology was performed via Ovid in MEDLINE, EMBASE and CENTRAL databases until July 31st, 2025, according to the Preferred Reporting Items for Systematic Reviews and Meta-analyses (PRISMA) guidelines (Fig. 1) [16].

Randomized controlled trials and retrospective or prospective observational studies including patients managed for infrarenal AAA (iAAA) with standard EVAR or managed for complex aortic aneurysm (cAA), including, juxta-, para-, suprarenal and TAAAs, and treated with complex EVAR [fenestrated/branched endovascular aortic repair (f/bEVAR)] were considered eligible. Studies should have been published in English, between January 1st, 2015, and July 31st, 2025, and assessing the impact of the pre-operative maximum aortic aneurysm diameter on 30-day and follow-up mortality. For f/bEVAR, both custom-made devices (CMD) and off-the-shelf aortic endografts could have been used. The application of parallel grafts (chimney technique) or physician modified endografts (PMEG) was not a criterion for exclusion, and these patients were included among the complex EVAR cases. Iliac branched devices were not used as criterion to define the complexity of the repair.

Studies reporting on patients managed with open surgical repair as an index procedure were excluded. Studies providing mixed populations (open and endovascular repair) that did not permit the safe data extraction for the endovascularly treated subgroup of the cohort were excluded. Patients managed endovascularly for lesions of the ascending aorta, aortic arch or solely the descending thoracic aorta, without extension of the repair into the renovisceral segment, were excluded. Case reports and series with less than 20 patients were not considered eligible.

### 2.2 Search Strategy 

The PICO [Patient; Intervention; Comparison; Outcome (Table 1)] model was followed [17].

**Table 1.** **The PICO model was applied to identify studies reporting on the outcomes of mortality after standard and complex EVAR**.

P	Patient, population or problem	Patients with infra-renal abdominal aortic aneurysm (iAAA) and complex aortic aneurysms (cAA), including juxta, para-, supra-renal and thoracoabdominal aortic aneurysms, managed with standard or complex endovascular aortic aneurysm repair, respectively
I	Intervention, prognostic factor or exposure	Pre-operative maximum aortic aneurysm diameter as a factor affecting mortality
C	Comparison of intervention	None
O	Outcome to be measured	Mortality at 30-days and beyond after standard and complex endovascular aortic aneurysm repair
	What type of question is asked?	Does the pre-operative maximum aortic aneurysm diameter affect the mortality at 30-days and follow-up after standard and complex endovascular aortic aneurysm repair?
T	Type of studies to be included	Randomized control trials, retrospective and prospective observational case-control and cohort studies

PICO, Patient, Intervention, Comparison, Outcome; EVAR, endovascular aortic aneurysm repair.

To identify any studies of interest, the following search terms (Table 2) of the Expanded Medical Subject Headings (MeSH) and keywords were used in various combinations: “abdominal aortic aneurysm”, “complex aortic aneurysm” OR “thoracoabdominal aneurysm” AND “endovascular aortic repair”, OR “EVAR”, OR “fenestrated endovascular aortic repair” OR “branched endovascular aortic repair” OR “f/bEVAR”, AND “mortality”, OR “survival”. A primary selection of relevant studies was based on title and abstract while a secondary selection was performed after assessing the full text of manuscript (P.N. and G.A.). Discrepancies were resolved after discussion with a third author (T.K.).

**Table 2.** **Search strategy of a systematic review focusing on the impact of the pre-operative aortic diameter on mortality after standard and complex endovascular aortic repair at 30 days and mid-term follow-up**.

Frame	Search terms (used both as full text and “MeSH” terms)	Search	Inclusion criteria	Exclusion criteria	Sources
P (population)	#1 “abdominal aortic aneurysm” OR #2 complex aortic aneurysm OR #3 “thoracoabdominal aneurysm”	#1 OR #2 OR #3 AND #4 OR #5 OR #6 OR #7 AND #8 AND #9 OR #10	Retrospective or prospective observational cohort studies and randomized control trials reporting on the impact of pre-operative aortic diameter on mortality after endovascular aortic repair	Irrelevant title or full text	PubMed, EMBASE, CENTRAL
I (intervention)	#4 “endovascular aortic aneurysm repair” OR #5 “EVAR” OR #6 “fenestrated endovascular aortic repair” OR #6 “branched endovascular aortic repair” OR #7 “f/bEVAR”		Peer reviewed journals	Editorial reviews, meta-analyses, case reports or series <20 patients	
C (comparator, reference test)	#8 “aortic diameter”		English language	Studies on open or hybrid aortic repair or with non-extractable data for the outcome of interest	
O (outcome)	#9 “mortality” OR #10 “survival”				
Time	Search period: 2015–2025				
	Last search: 31.07.2025				

MeSH, Medical Subject Headings; f/bEVAR, fenestrated/branched endovascular aortic repair.

### 2.3 Data Extraction 

A standardized extraction Microsoft Excel file was developed by two authors (P.N. and G.A.). Extracted data included study characteristics (authors, journal, year of publication, study design, timespan, country/center/database) in addition to general information [number of patients, baseline demographics (age, sex)], underlying disease [iAAA and cAA (juxta-, para-and supra-renal aneurysms or TAAA)] and type of repair [EVAR or complex EVAR (fbEVAR, chimney EVAR)]. The pre-operative aortic aneurysm diameter thresholds for patients’ stratification into small and large aortic aneurysms were collected, when available. The post-operative mortality at 30-days and follow-up was noted, as well as the role of the pre-operative maximum aortic aneurysm diameter on mortality, when assessed through multivariate or propensity matched analyses. No attempt was made to retrieve missing data from the authors of the included studies.

### 2.4 Quality Assessment 

The risk of bias was assessed using the Newcastle-Ottawa scale, which evaluates observational studies using a star system through three methodological domains: selection, comparability and outcomes [18]. The assessment using the Newcastle-Ottawa scale was performed by two independent authors (P.N. and G.A.). Discrepancies were resolved after discussion with a third author (T.K.).

### 2.5 Outcomes

The impact of the pre-operative maximum aortic aneurysm diameter on 30-day mortality after standard and complex EVAR was the primary outcome. Secondary was the impact of the pre-operative maximum aortic diameter on standard and complex EVAR mortality during follow-up.

### 2.6 Statistical Analysis 

This is a narrative review of the available literature and only descriptive data is provided. No meta-analytic assessment or comparisons was attempted due to the heterogeneity on aortic aneurysm diameter thresholds used to define smaller vs. larger aortic aneurysm. The heterogeneity was met in both studies describing standard and complex EVAR. In addition, the studies reporting on complex EVAR included a variety of techniques and aneurysms’ extents; another parameter hampering a safe data synthesis.

## 3. Results

### 3.1 Study Selection

The initial systematic search retrieved 1182 studies. After applying the predefined inclusion criteria, 25 were selected (Fig. 1); nineteen reporting on the role of the pre-operative maximum aortic aneurysm diameter on standard EVAR mortality and six on complex EVAR mortality. All studies were observational, and of retrospective design. The studies’ main characteristics are shown in Table 3 (Ref. [18, 19, 20, 21, 22, 23, 24, 25, 26, 27, 28, 29, 30, 31, 32, 33, 34, 35, 36]) for standard EVAR and Table 4 (Ref. [14, 37, 38, 39, 40, 41]) for complex EVAR [14, 18, 19, 20, 21, 22, 23, 24, 25, 26, 27, 28, 29, 30, 31, 32, 33, 34, 35, 36, 37, 38, 39, 40, 41]. Seventeen studies were of comparative design; fourteen reporting on standard EVAR and three on complex EVAR [14, 18, 19, 20, 22, 23, 24, 25, 27, 28, 29, 30, 31, 34, 35, 40, 41]. The remaining studies evaluated the pre-operative maximum aortic aneurysm diameter as a factor affecting mortality only through multivariate or propensity matched analyses [21, 26, 32, 33, 36, 37, 38, 39].

**Table 3.** **Main characteristics of the included studies reporting on the impact of the pre-operative aortic iAAA diameter on standard EVAR mortality at 30 days and follow-up [18, 19, 20, 21, 22, 23, 24, 25, 26, 27, 28, 29, 30, 31, 32, 33, 34, 35, 36]**.

Author	Year	Journal	Country	Database	Design	Timespan of data collection
Scali *et al*. [18]	2022	J Vasc Surg	USA	VQI	Retrospective cohort	2015–2019
Oliveira *et al*. [19]	2019	J Vasc Surg	Netherlands	Single center	Retrospective cohort	2000–2014
Fan *et al*. [20]	2023	J Vasc Surg	USA	VQI	Retrospective cohort	2010–2020
Antoń *et al*. [21]	2024	Ren Fail.	Poland	Single center	Retrospective cohort	NA
Jones *et al*. [22]	2019	J Vasc Surg	USA	VQI	Retrospective cohort	2003–2017
Wiatrzyk *et al*. [23]	2025	J Clin med	Poland	Single center	Retrospective cohort	2016–2024
Kim *et al*. [24]	2021	J Surg Res.	USA	VQI	Retrospective cohort	2003–2018
Jeon-Slaughter *et al*. [25]	2019	J Endovasc Ther	USA	Single center	Retrospective cohort	2003–2013
Rašiova *et al*. [26]	2023	VASA	Slovakia	Single center	Retrospective cohort	2010–2019
Sirignano *et al*. [27]	2020	Ann Vasc Surg	Italy	Single center	Retrospective cohort	2008–2015
de Guerre *et al*. [28]	2021	J Vasc Surg	USA	VQI	Retrospective cohort	2003–2016
de Guerre *et al*. [29]	2022	J Vasc Surg	USA	VQI	Retrospective cohort	2003–2016
Huang *et al*. [30]	2017	J Vasc Surg	USA	Single center	Retrospective cohort	1997–2011
Ramos *et al*. [31]	2020	Vasc Endovasc Surg	USA	NSQIP	Retrospective cohort	2011–2015
Fitridge *et al*. [32]	2016	Eur J Vasc Endovasc Surg	Australia	Two Australia Audits	Retrospective cohort	1999–2001 and 2009–2013
Rašiova *et al*. [33]	2025	Vascular	Slovakia	Single center	Retrospective cohort	2010–2021
Hye *et al*. [34]	2019	Ann Vasc Surg	USA	Integrated health system’s AAA endograft registry	Retrospective cohort	2010–2014
Davis *et al*. [35]	2019	J Vasc Surg	USA	Statewide vascular surgery registry	Retrospective cohort	2012–2016
Ünal *et al*. [36]	2021	Kardiochir Torakochirurgia Pol	Türkiye	Single center	Retrospective cohort	2013–2019

Footnotes: AAA, abdominal aortic aneurysm; NSQIP, National Surgical Quality Improvement Program; VQI, Vascular Quality Initiative; NA, non-applicable.

**Table 4.** **Main characteristics of the included studies reporting on the impact of the pre-operative aortic aneurysm diameter on complex EVAR mortality at 30 days and follow-up [14, 37, 38, 39, 40, 41]**.

Author	Year	Journal	Country	Database	Design	Timespan of data collection
Arnaoutakis *et al*. [37]	2024	J Vasc Surg	USA	VQI	Retrospective cohort	2012–2023
Elizaga *et al*. [14]	2025	Eur J Vasc Endovasc Surg	USA	VQI	Retrospective cohort	2013–2022
Banks *et al*. [38]	2024	J Vasc Surg	USA	US-ARC	Retrospective cohort	2005–2022
van Calster *et al*. [39]	2019	J Vasc Surg	USA	Single center	Retrospective cohort	2004–2016
van Galen *et al*. [40]	2025	J Vasc Surg	USA	VQI	Retrospective cohort	2012–2024
Gallitto *et al*. [41]	2024	Eur J Cardiothor Surg.	Italy	Two-center	Retrospective cohort	2011–2021

Footnotes: US-ARC, US Aortic Research Consortium.

### 3.2 Risk of Bias 

The Newcastle-Ottawa Scale assessment revealed significant bias among the included studies and categorized thirteen as poor quality (52%) [19, 21, 26, 30, 31, 32, 33, 34, 35, 36, 37, 39, 40], four as fair quality (16%) [23, 25, 27, 38], and the remaining eight (32%) as good quality [14, 18, 20, 22, 24, 28, 29, 41]. Bias was mainly attributed to the retrospective nature, lack of comparative arm, inadequate reporting of confounding factors and management of missing data (Table 5, Ref. [14, 18, 19, 20, 21, 22, 23, 24, 25, 26, 27, 28, 29, 30, 31, 32, 33, 34, 35, 36, 37, 38, 39, 40, 41]).

**Table 5.** **Risk of bias assessment of the included studies using the Newcastle Ottawa Scale**.

Author	Selection	Comparability	Exposure	Risk of bias (total number of stars)
Standard EVAR				
Scali *et al*. [18]	***	*	**	6
Oliveira *et al*. [19]	**	*	*	4
Fan *et al*. [20]	***	*	**	6
Antoń *et al*. [21]	*	NA	*	3
Jones *et al*. [22]	***	*	**	6
Wiatrzyk *et al*. [23]	**	*	**	5
Kim *et al*. [24]	***	*	**	6
Jeon-Slaughter *et al*. [25]	**	*	**	5
Rašiova *et al*. [26]	*	NA	**	3
Sirignano *et al*. [27]	**	*	**	5
de Guerre *et al*. [28]	***	*	**	6
de Guerre *et al*. [29]	***	*	**	6
Huang *et al*. [30]	**	*	**	5
Ramos *et al*. [31]	***	*	*	5
Fitridge *et al.* [32]	*	NA	**	3
Rašiova *et al*. [33]	*	NA	**	3
Hye *et al*. [34]	***	*	*	5
Davis *et al*. [35]	***	*	*	5
Ünal *et al*. [36]	*	NA	*	2
Complex EVAR				
Arnaoutakis *et al*. [37]	**	NA	*	3
Elizaga *et al*. [14]	***	*	**	6
Banks *et al*. [38]	***	*	*	5
van Calster *et al*. [39]	**	NA	*	3
van Galen *et al*. [40]	**	NA	**	4
Gallitto *et al*. [41]	***	*	**	6

Footnotes: NA, non-applicable.

### 3.3 Study Cohort and Pre-Operative Aortic Diameter Thresholds

In total, 130,476 patients were included in the standard EVAR cohort; 28,226 (29.5%) were categorized as patients with larger iAAA, according to the aortic aneurysm diameter thresholds provided in each study (Table 6, Ref. [18, 19, 20, 21, 22, 23, 24, 25, 26, 27, 28, 29, 30, 31, 32, 33, 34, 35, 36]). Eighteen studies reported on intact iAAA [18, 19, 20, 22, 23, 24, 25, 26, 27, 28, 29, 30, 31, 32, 33, 34, 35, 36]; fourteen of them exclusively on electively managed cases [18, 20, 22, 26, 27, 28, 29, 30, 31, 32, 33, 34, 35, 36]. Only one study, by Antoń *et al*. [21], reported selectively on ruptured iAAA. A significant heterogeneity was recorded regarding the diameter thresholds applied to identify a larger vs. smaller aneurysm. Eight studies used the diameter threshold (≥55 mm for males and ≥50 mm for females) reported in the recent abdominal aortic aneurysm recommendations [42] and targeted to verify the outcomes of EVAR in patients managed with smaller aneurysms [18, 20, 22, 24, 29, 31, 34, 35]. In three out of these studies, a separate diameter threshold; set at 50 mm, was used for the female population (Table 6) [20, 29, 35].

**Table 6.** **Patient cohorts and pre-operative aortic aneurysm diameter thresholds applied per study to stratify the population and investigate the impact of diameter on standard EVAR mortality**.

Author	Number of patients	Patients with a larger aneurysm	Diameter threshold to identify large aneurysms	Setting of repairs
Scali *et al*. [18]	25,112	9675	≥55 mm	Elective
Oliveira *et al*. [19]	404	86	>70 mm	Intact
Fan *et al*. [20]	1974	309	≥55 mm for males, ≥50 mm for females	Elective
Antoń *et al*. [21]	192	NR	>67 mm	Ruptured
Jones *et al*. [22]	22,975	2780	≥65 mm	Elective
Wiatrzyk *et al*. [23]	176	59	≥64 mm	Intact
Kim *et al*. [24]	32,398	NR	≥55 mm	Intact
Jeon-Slaughter *et al*. [25]	325	141	≥56 mm and ≥60 mm	Intact
Rašiova *et al*. [26]	162	NR	NR	Elective
Sirignano *et al*. [27]	498	107	≥59 mm	Elective
de Guerre *et al*. [28]	16,289	2729	≥65 mm	Elective
de Guerre *et al*. [29]	19,018	6730	≥55 mm for males, ≥50 mm for females	Elective
Huang *et al*. [30]	874	266	≥60 mm	Elective
Ramos *et al*. [31]	2115	901	≥55 mm	Elective
Fitridge *et al*. [32]	1647	NR	≥65 mm	Elective
Rašiova *et al*. [33]	196	NR	NR	Elective
Hye *et al*. [34]	1967	996	≥55 mm	Elective
Davis *et al*. [35]	3932	3447	≥55 mm for males, ≥50 mm for females	Elective
Ünal *et al*. [36]	222	NR	≥60 mm	Elective

Footnotes: NR, not reported.

Regarding complex EVAR studies, 14,097 patients were included; all managed under elective setting [14, 37, 38, 39, 40, 41]. Among them, 1433 (19.2%) were classified as having a larger cAA [14, 40, 41]. Three studies reported exclusively on f/bEVAR cases [38, 39, 41]. A similar heterogeneity, as in the standard EVAR studies, was detected in terms of diameter thresholds applied to identify a larger vs. smaller cAA. However, three studies suggested a threshold over 70 mm, with one of them setting the threshold over 80 mm [14, 38, 41]. One study applied the suggested diameter threshold for repair according to the Society of Vascular Surgery guidelines (≥55 mm for complex abdominal aortic aneurysms) [37, 42], and one set a threshold ≥65 mm for males and ≥60 mm for females [40]. One study did not report on a specific threshold but investigated the role of the pre-operative aortic aneurysm diameter on mortality after complex EVAR (Table 7, Ref. [14, 37, 38, 39, 40, 41]) [39].

**Table 7.** **Patient cohorts and pre-operative aortic diameter thresholds applied per study to stratify the population and investigate the impact of aortic aneurysm diameter on complex EVAR mortality**.

Author	Number of patients	Patients with a larger aneurysm	Diameter threshold to identify large aneurysms	Setting of repairs	Type of repair
Arnaoutakis *et al*. [37]	4053	NR	≥55 mm	Elective	f/bEVAR, chEVAR
Elizaga *et al*. [14]	3804	600	>70 mm	Elective	f/bEVAR with PMEG
Banks *et al*. [38]	2099	NR	>70 mm	Elective	f/bEVAR
van Calster *et al*. [39]	468	NR	>67 mm	Elective	f/bEVAR
van Galen *et al*. [40]	3426	774	≥65 mm for males and ≥60 mm for females	Elective	f/bEVAR, chEVAR/parallel graft
Gallitto *et al*. [41]	247	59	≥80 mm	Elective	f/bEVAR

Footnotes: chEVAR, chimney endovascular aortic aneurysm repair; PMEG, physician modified endografts.

### 3.4 Association of the Pre-Operative Aortic Aneurysm Diameter With the 30-Day and Follow-Up Mortality in Standard EVAR Studies

Eight out of nineteen studies evaluated the impact of the pre-operative iAAA diameter on the 30-day mortality after standard EVAR [20, 22, 25, 29, 30, 31, 34, 35]. Among these eight studies, four concluded to a statistically significant difference in 30-day mortality, with patients managed for a smaller iAAA presenting lower mortality rates (Table 8, Ref. [18, 19, 20, 21, 22, 23, 24, 25, 26, 27, 28, 29, 30, 31, 32, 33, 34, 35, 36]) [20, 22, 29, 34]. The remaining four studies failed to show a difference in 30-day mortality when comparing patients with smaller vs. larger iAAA [25, 30, 31, 35]. The range for mortality in patients with smaller iAAA was 0.3–13.2% vs. 0.7–20.8% in patients with larger iAAA [20, 22, 25, 29, 30, 31, 34, 35]. Five studies evaluated through further analyses the potential independent role of the pre-operative maximum aortic diameter on the 30-day mortality of standard EVAR; four through multivariate logistic and cox-regression [30, 31, 32, 34], while one using a propensity matched analysis [36]. In two studies, the pre-operative maximum iAAA diameter was identified as independent predictor for 30-day mortality after standard EVAR for iAAA [32, 33].

**Table 8.** **The impact of the pre-operative aortic diameter on standard EVAR mortality as assessed in the included studies**.

Author	30-day mortality in smaller aneurysms	30-day mortality in larger aneurysms	Pre-operative aortic diameter as predictor for 30-day mortality	Follow-up mortality/survival in smaller aneurysms	Follow-up mortality/survival in larger aneurysms	Pre-operative aortic diameter as predictor for follow-up mortality
Scali *et al*. [18]	NR	NR	NR	92 ± 2%	97 ± 1%	NR
Oliveira *et al*. [19]	NR	NR	NR	NR	NR	HR, 1.75; 95% CI, 1.20–3.56
Fan *et al*. [20]	0.3%	0.8%	NR	87.6%	80.7%	NR
Antoń *et al*. [21]	NR	NR	NR	31.6% of dead patients had iAAA >67 mm vs. 21.8% of alive patients had iAAA >67 mm	NR	NR
Jones *et al*. [22]	0.4%	1.6%	NR	88%	75%	HR, 1.50; *p * < 0.001
Wiatrzyk *et al*. [23]	NR	NR	NR	NR	NR	Not independently related
Kim *et al*. [24]	NR	NR	NR	NR	NR	HR, 0.66; 95% CI, 0.61–0.72; *p * < 0.01
Jeon-Slaughter *et al*. [25]	1.1%	2.1%	NR	NR	NR	Not independently related
Rašiova *et al*. [26]	NR	NR	NR	NR	NR	HR, 1.07; 95% CI, 1.03–1.12; *p* = 0.001 (diameter as continuous variable) and HR, 2.23; 95% CI, 1.18–4.24; *p* = 0.014 (threshold over 55 mm)
Sirignano *et al*. [27]	NR	NR	NR	NR	NR	OR, 4.00; 2.46–6.49; *p * < 0.001
de Guerre *et al*. [28]	NR	NR	NR	NR	NR	HR, 1.52; 95% CI, 1.40–1.67; *p * < 0.001
de Guerre *et al*. [29]	0.7%	1.6%	NR	71%	61%	HR, 1.6; 95% CI, 1.4–1.7; *p * < 0.001
Huang *et al*. [30]	1.9%	1.7%	Not independently related	44 ± 6%	25 ± 9%	HR, 2.33; 95% CI, 1.64–3.32; *p * < 0.001
Ramos *et al*. [31]	0.5%	0.7%	Not independently related	NR	NR	NR
Fitridge *et al*. [32]	NR	NR	OR, 0.64; *p * < 0.001	NR	NR	NR
Rašiova *et al*. [33]	NR	NR	NR	NR	NR	HR, 1.05; 95% CI, 1.03–1.08; *p * < 0.001
Hye *et al*. [34]	13.2%	20.8%	HR, 1.94; 95% CI, 1.32–2.86	NR	NR	NR
Davis *et al*. [35]	1.8%	2.1%	After propensity matching, *p* = 0.35	5.9%	7.2%	After propensity matching, *p* = 0.99
Ünal *et al*. [36]	NR	NR	NR	NR	NR	HR, 2.20; 95% CI, 1.01–4.81; *p* = 0.049

Footnotes: CI, confidence interval; HR, hazard ratio; NR, not reported; OR, odds ratio.

Regarding the follow-up outcomes (Table 8), five studies showed a statistically significant difference of higher mortality in patients with larger iAAA [20, 21, 22, 29, 30] while the study by Davis *et al*. [35] suggested that the follow-up mortality was equal between patients with smaller vs. larger iAAA. The study by Scali *et al*. [18] reported that EVAR-treated patients with a diameter below the guideline-suggested threshold of 55 mm (non-guideline-compliant EVAR) presented with a worse 1-year survival compared to patients treated within the recommended diameter thresholds (92 ± 2% vs. 97 ± 1%; *p* < 0.0001). Nine studies showed that the pre-operative maximum aortic diameter was independently related to mortality after standard EVAR and increased the risk for mortality by 2.4 times [19, 22, 26, 27, 28, 29, 30, 33, 36]. In one study, by Sirignano *et al*. [27], the follow-up mortality was not assessed as time-to-event analysis and the provided significance was reported through a multivariate logistic analysis. Two studies failed to identify an independent correlation of the pre-operative maximum iAAA diameter to follow-up mortality [23, 25].

### 3.5 Association of the Pre-Operative Aortic Aneurysm Diameter With the 30-Day and Follow-Up Mortality in Complex EVAR Studies 

Four out of six studies reported on the 30-day mortality after complex EVAR (Table 9, Ref. [14, 37, 38, 39, 40, 41]). Three out of these studies detected a significant difference in mortality in patients with larger aneurysms [14, 39, 40] while one showed non-significant difference [41]. The mortality range in patients with smaller cAA was 0.5–7.0% vs. a mortality ranging between 4.0–15.0% in patients with larger cAA [14, 39, 40, 41]. In three studies, the pre-operative aortic diameter was identified as an independent predictor for 30-day mortality after complex EVAR [14, 39, 40].

**Table 9.** **The impact of the pre-operative aortic diameter on complex EVAR mortality as assessed in the included studies**.

Author	30-day mortality in smaller aneurysms	30-day mortality in larger aneurysms	Pre-operative aortic diameter as predictor for 30-day mortality	Follow-up mortality/survival in smaller aneurysms	Follow-up mortality in larger aneurysms	Pre-operative aortic diameter as predictor for follow-up mortality
Arnaoutakis *et al*. [37]	NR	NR	NR	13%	5%	NR
Elizaga *et al*. [14]	1.0%	4.0%	OR, 3.43; 95% CI, 1.43–10.4	87%	65%	HR, 2.4; 95% CI, 1.6–3.7
Banks *et al*. [38]	NR	NR	NR	NR	NR	HR, 1.68; 95% CI, 1.28–2.21
van Calster *et al*. [39]	NR	NR	OR, 1.053 per millimeter; 95% CI, 1.020–1.087	NR	NR	HR, 1.053 per millimeter; 95% CI, 1.020–1.087
van Galen *et al*. [40]	0.5%	4.8%	aOR, 1.73; 95% CI, 1.09–2.72	NR	NR	HR, 1.50; 95% CI, 1.19–1.88
Gallitto *et al*. [41]	7.0%	15.0%	Not independently related	NR	NR	Not independently related

Footnotes: aOR, adjusted odds ratio.

Regarding the follow-up outcomes, one study showed a statistically significant difference of higher mortality in larger cAA [14] while the study by Arnaoutakis *et al*. [37] suggested that the follow-up mortality is higher in patients presenting with an aneurysm below the diameter threshold suggested by the guidelines. In four studies, the pre-operative aortic diameter was detected as a factor affecting independently the follow-up mortality and increased the mortality risk by 1.05 to 2.4 times [14, 38, 39, 40]. Only the study by Gallitto *et al*. [41] reported that the pre-operative diameter was not related to worse mortality during follow-up.

## 4. Discussion 

Aortic aneurysm anatomy has been previously related to the technical and clinical outcomes of EVAR, regardless the extent of the disease [43, 44, 45, 46]. Among other anatomic factors, such as the proximal and distal landing zone, the pre-operative aortic aneurysm diameter seems, also, to play a significant role in outcomes, such as mortality, morbidity, endoleak evolution and reintervention at 30 days and during further follow-up [13, 27, 28, 29, 30]. In this systematic review, the role of the pre-operative maximum aortic diameter on the 30-day mortality after standard EVAR for iAAA seems to be unclear, with 50% of the included studies and 40.7% of the patients leading to a non-statistically significant finding [20, 22, 25, 29, 30, 31, 34, 35]. The role of the pre-operative maximum aortic diameter was more indicative of mortality during follow-up, with ten vs. two studies showing an independent correlation in standard EVAR cohorts [19, 22, 24, 26, 27, 28, 29, 30, 33, 36]. In complex EVAR, the pre-operative maximum aortic diameter impact seems to be more prominent both regarding the 30-day and follow-up mortality [14, 38, 39, 40].

Current recommendations suggest endovascular repair in patients with suitable anatomy that exceed a diameter threshold of 55 mm in males and 50 mm in females for iAAA and complex abdominal aortic aneurysms; including juxta- and pararenal cases [1, 42]. TAAA repair is suggested when the diameter exceeds 60 mm, regardless patients’ sex [47]. However, the low mortality rates, especially after standard EVAR, raised concerns regarding the potential application of even lower iAAA diameter thresholds for repair [20, 22]. As shown in this systematic review, among the six studies that evaluated the application of EVAR in patients with smaller diameter thresholds, the outcomes were inconclusive regarding the impact on mortality, with 50% of the studies showing no difference [18, 31, 35]. Only one study, by Fan *et al*. [20], identified an aortic diameter below the suggested thresholds as a predictor for better survival while the study by Scali *et al*. [18] suggested a lower survival in patients managed with EVAR for iAAA below 55 mm. However, previous studies reporting exclusively on ruptured aortic aneurysms showed that ruptures may occur in smaller aortic diameters, especially in females, while patients managed for a ruptured small aneurysm may present a survival benefit [48, 49]. For complex EVAR, the available data is very scarce, with only one study evaluating the guideline-suggested aortic diameter threshold for repair [37]. This study showed that patients managed at diameters lower than the suggested threshold had significantly worse survival after adjusting for confounders [37]. More studies are needed to evaluate the role of aortic diameter on cAA outcomes, and especially, TAAAs [14].

For standard EVAR studies, the heterogeneity on the applied diameter thresholds to identify any potential impact of mortality is remarkable. However, when combining the findings shown in Table 6 and Table 8 and evaluating only the studies setting a diameter threshold over 60 mm for iAAA, the pre-operative diameter was detected as an independent factor affecting mortality during follow-up in seven (78%) out of nine cohorts [19, 21, 22, 23, 25, 28, 30, 32, 36]. This could set a question of an optimal aortic diameter range to provide standard EVAR in iAAA patients, by respecting the lower threshold suggested by the available guidelines (55 mm for males and 50 mm for females) and offer a stricter surveillance to patients with iAAA diameter close to the diameter threshold for repair while proceeding with the procedure soon after confirming the diagnosis; before a further aneurysm enlargement affects EVAR clinical outcomes. Current evidence shows that an increase of 5 mm could lead to worse outcomes in standard EVAR cases, reflecting the risks of a delayed surgical response.

For complex EVAR mortality, the pre-operative diameter seems to have a more clear role on mortality after elective repair of cAA, with three out of four (75%) studies that assessed the pre-operative aortic diameter through multivariate analyses, identifying it as an independent predictor at 30-days [14, 39, 40] while four out of five (80%) detected the pre-operative aortic diameter as a predictor for follow-up mortality [14, 38, 39, 40]. The diameter threshold over 65 mm seems to be meaningful in this heterogenic cohort of complex abdominal aortic aneurysms and TAAAs, with 70 mm providing an even clearer correlation between mortality and pre-operative aortic diameter after complex EVAR [14]. In the study by Elizaga *et al*. [14], a subanalysis focusing on complex abdominal aortic aneurysms vs. TAAAs still showed that a diameter over 70 mm was related to higher mortality, regardless the extend of the disease; a parameter that has been multiple times identified as predictor for worse outcomes after endovascular and open repair, especially regarding type II TAAAs [11, 50, 51].

The mechanism justifying the higher mortality in patients with larger aortic aneurysms managed endovascularly may be related to the presence of more progressed disease, as larger aneurysm diameter has been related to higher risk for rupture and more hostile proximal aortic neck anatomy [52]. In addition, the aortic diameter has been shown to relate with the presence of other cardiovascular risk factors, including ischemic heart disease and chronic kidney disease; with rates up to two times higher in patients with an aortic diameter over 35 mm [53, 54]. The incidence of cardiovascular events seems to be even more prominent in female patients with larger infrarenal aortic diameters; a fact that may explain the worse EVAR outcomes in the female population and justify the need to re-evaluate the diameter thresholds for repair applied in females, using indicators other than aortic diameter [55]. Ongoing multinational randomized controlled trials, such as the Women’s Aneurysm Research: Repair Immediately or Routine Surveillance (WARRIORS) trial, even aim to determine the optimal aortic diameter threshold for women with iAAA, as previous trial cohorts included only 5% of females.

Despite that this systematic review focused on mortality after standard and complex EVAR, studies showed that the pre-operative diameter may also be predictive for technical success and reinterventions, as well as clinical outcomes, including major adverse events [30, 56, 57]. Larger diameter aneurysms may complicate the technical completion of standard EVAR cases, as their presence is related to worse proximal and distal neck characteristics and thrombus presence, while they may be related to more challenging target vessel bridging and further, instability events in complex repair using f/bEVAR [56]. In addition, aortic sac behavior after endovascular repair, even in successful cases with sac regression, may lead to endograft and/or bridging stent deformation and higher reintervention rates during follow-up, especially in cases of large cAA managed with f/bEVAR, where the aneurysm sac may show a more dramatic size decrease [41, 58, 59].

Considering the information discussed above, the pre-operative aortic aneurysm diameter, regardless the extend of the disease, seems to be the expression of a multifactorial background that may lead to worse survival in patients with infrarenal or more complex aortic aneurysmal disease [52, 56, 57]. Patients with larger aortic aneurysms seem to carry a more demanding anatomic profile, including hostile proximal and distal sealing zones and target vessel and access anatomy, which may hamper a technically successful intervention and increase the risk for reintervention [56, 60]. The presence of higher rate of comorbidities in these patients with larger aortic aneurysm is rather a reflection of an advance vascular disease, which affects more than one beds and leads to decreased survival during follow-up. The common underlying factors, including tobacco consumption, hypertension and hyperlipidemia, create the “adequate” environment for the evolution of aortic aneurysms and atherosclerotic disease affecting both the peripheral and coronary arteries [61].

### Limitations 

The limited number of studies, especially for the impact of the pre-operative maximum aortic diameter on complex EVAR mortality, in addition to the retrospective nature of all included studies introduced significant bias due to confounders and should be acknowledged upon interpretation of the findings of this systematic review. A significant heterogeneity on the pre-operative aortic diameter thresholds cannot provide for the moment a common acceptable diameter that could be assessed as a factor affecting mortality. In addition, a few studies, especially, from the standard EVAR group, compared patients who were treated without reaching the diameter threshold, raising valid questions about the application of EVAR in a population that may not benefit from repair [42]. The extend of the disease was not assessed, while it has been shown before to constitute a parameter that impacts the post-operative mortality and morbidity, while, especially in the complex EVAR studies, a wide heterogeneity of techniques and disease patterns were included. This potentially affected the findings of the current review and highlights the gap existing in the available literature, regarding the role of the pre-operative maximum aortic diameter on standard and complex EVAR cases. The limitations mentioned above, including the lack of standardized diameter threshold to define smaller vs. larger aortic aneurysms, in addition to the various techniques and aneurysms’ extent, do not permit for the moment a safe data synthesis using a metanalytic approach. Thus, a descriptive approach was chosen for the current review, in order not only to show the available literature but further highlight the lack of robust evidence. The exclusion of smaller observational studies potentially affected the findings of the current review.

## 5. Conclusions

According to the current systematic review, the pre-operative aortic aneurysm diameter may be predictive of 30-day mortality in patients managed either with standard or complex EVAR. However, the impact of diameter on mortality after complex EVAR may be more prominent, as 80% of the studies confirm this finding. Regarding follow-up, the pre-operative aortic diameter is predictive for mortality both in standard as well as complex EVAR cases. The heterogeneity on diameter thresholds to stratify cases as larger aneurysms hampers for the moment a data synthesis and should be taken into consideration when interpreting the findings of this review.

## Data Availability

The data are available from the corresponding author after reasonable demand.

## Abbreviations

AAA, abdominal aortic aneurysm; cAA, complex aortic aneurysm; CTA, computed tomography angiography; EVAR, endovascular aortic aneurysm repair; f/bEVAR, fenestrated/branched endovascular aortic repair; iAAA, infrarenal abdominal aortic aneurysm; TAAA, thoracoabdominal aneurysm.

## Supplementary Material

Supplementary material associated with this article can be found, in the online version, at https://doi.org/10.31083/RCM47199.
